# The Impact of Long COVID on Language Proficiency Across Different School Levels in Hong Kong

**DOI:** 10.3390/bs15040432

**Published:** 2025-03-28

**Authors:** Shebe S. Xu, Yixun Li, Wanyi Li, Catherine M. Capio, Winnie W. Y. Tso, Derwin K. C. Chan

**Affiliations:** 1 Department of Early Childhood Education, Faculty of Education and Human Development, The Education University of Hong Kong, 10 Lo Ping Road, Tai Po, New Territories, Hong Kong; siwei_xu@yeah.net (S.S.X.); lyixun@eduhk.hk (Y.L.); 2 Department of Curriculum and Instruction, The Chinese University of Hong Kong, Hong Kong; wanyi_li@outlook.com; 3 Department of Physiotherapy, Hong Kong Metropolitan University, Hong Kong; ccapio@hkmu.edu.hk; 4 Health Science Department, Ateneo de Manila University, Quezon City 1108, Philippines; 5 Department of Pediatrics and Adolescent Medicine, School of Clinical Medicine, The University of Hong Kong, Hong Kong; wytso@hku.hk

**Keywords:** long COVID, COVID-19, language ability, children and adolescents, speaking, listening

## Abstract

Long COVID, where symptoms persist after recovering from COVID-19, can affect cognitive functions like language. However, little is known about its impact on children’s language skills, especially across different school levels. This study investigated the impact of long COVID on language proficiency among 1244 children (Asian; 53.5% boys) from kindergartens (*N* = 408, *M_age_* = 4.42 ± 1.26 years), primary schools (*N* = 547, *M_age_* = 9.69 ± 1.96 years), and secondary schools (*N* = 289, *M_age_* = 14.97 ± 1.85 years) in Hong Kong. Language proficiency was assessed using the Language Experience and Proficiency Questionnaire (LEAP-Q), which measured speaking, listening, reading, and writing in both Chinese and English. Participants were categorized into three groups: long COVID, recovered from COVID-19, and no history of COVID-19. One-way and two-way ANOVAs were used to analyze the differences in language proficiency across these groups and school levels. Children with long COVID symptoms exhibited significantly lower overall language proficiency, particularly in speaking and listening, compared to those in the recovered and no-COVID groups. The effect was more pronounced among primary and secondary students, with secondary school students showing the most substantial deficits. No significant differences were found between the recovered and no-COVID groups. The results suggest that long COVID might have detrimental effects on children’s linguistic proficiency. The language development of older students who suffered from long COVID could benefit from receiving targeted educational and therapeutic interventions.

## 1. Introduction

Empirical studies have extensively documented the enduring cognitive impairments observed in individuals post-COVID-19 infection and during the recovery phase, commonly known as “long-COVID” (Crivelli et al., 2022; Hadad et al., 2022; Hampshire et al., 2021; Raveendran et al., 2021). Long COVID, also known as post-COVID-19 syndrome, is a condition in which individuals who have recovered from COVID-19 continue to experience persistent symptoms such as heightened fatigue, cough, and even cognitive dysfunction, including deficits in attention, executive functioning, language, processing speed, and memory symptoms collectively referred to as “brain fog” (CDC, 2023; Crook et al., 2021). Long COVID symptoms are also related to increased anxiety, depression, sleep disorders, and fatigue (Venkataramani & Winkler, 2022). A recent meta-analysis and systematic review by Chen et al. (2022) further highlighted the global prevalence of post-COVID-19 conditions, estimating that approximately 43% of individuals infected with COVID-19 experience long-term health consequences. This underscores the significant burden of long COVID on public health.

Emerging evidence reveals that pediatric populations are not immune to these protracted effects. A recent cohort study of 5367 children and adolescents (6–17 years) identified 14 common symptoms persisting at least 4 weeks post-SARS-CoV-2 infection, including fatigue, cognitive difficulties, and gastrointestinal issues (Gross et al., 2024). Notably, younger children (6–11 years) exhibited unique symptoms such as sleep disturbances and abdominal pain, whereas adolescents (12–17 years) reported more pronounced cognitive impairments and emotional distress. While prior research has established COVID-19’s detrimental effects on communicative abilities in adults, including word-finding difficulties and reduced verbal fluency (Huang et al., 2021), direct evidence linking long COVID to language development in children remains scarce (Cummings, 2023, 2024). This study addresses this gap by comparing the language proficiency of children with long COVID symptoms, with that of children who have fully recovered from COVID-19 and those who have never been infected with COVID-19.

The pandemic’s dual impact—combining viral pathology with societal disruptions—has uniquely threatened developmental trajectories. The COVID-19 pandemic has been shown to have deleterious effects on children’s health, neurodevelopment, and cognitive performance (Deoni et al., 2021). Quarantine measures during the pandemic, such as social distancing, mandatory face masks, school suspensions, and lockdowns, have been suggested to have severe negative effects on the quality and volume of human communication (Charney et al., 2021; Moon et al., 2022; Najamuddin et al., 2022). Furthermore, COVID-19 infection itself may impair memory, attention, concentration, and executive function (Borch et al., 2022; Buonsenso et al., 2021; Ludvigsson, 2021). Recent research has indicated that COVID-19 can quickly impair cognitive functions, particularly verbal skills. Additionally, up to 11% of children who had COVID-19 show difficulties with word fluency (Beaud et al., 2021; Negrini et al., 2021). These findings suggest that the virus may directly impair specific linguistic abilities, highlighting the need for more in-depth and context-sensitive studies to fully understand the pandemic’s consequences (Hopp & Thoma, 2020).

Despite well-documented impairments of pandemic restrictions on children’s language development (Ahrens et al., 2021; Ganesan et al., 2021), there is limited research on how long COVID symptoms affect language skills. Existing studies have shown that a substantial proportion of adults who were diagnosed with long COVID symptoms experienced language difficulties, such as losing concentration, finding reading challenging, and mixing up or producing incorrect words (Cummings, 2022, 2023). However, findings from adult studies may not be generalizable to the pediatric population, highlighting a gap in current research. Given the critical role language plays in children’s academic, social, and cognitive development, it is essential to examine the effects of long COVID on children. Furthermore, it is important to contrast these impacts among different clusters, such as children with long COVID and those without. Comparing children with long COVID to those without, who have similarly experienced pandemic measures, can help determine whether language impairments are primarily due to long COVID symptoms or situational factors. The unique trajectory of COVID-19 in Hong Kong, characterized by strict early containment measures followed by widespread Omicron-variant outbreaks, provides a critical sociocultural context for understanding long COVID’s educational impacts. Prolonged school closures and reliance on online learning (February 2022–May 2023) disrupted structured language instruction, while the resumption of in-person classes under strict mitigation measures (e.g., mandatory masking, reduced social interaction) constrained communicative environments (So et al., 2022). Coupled with the city’s bilingual education system and densely urban population, these conditions created a natural experiment for examining how pandemic-related disruptions interact with long COVID’s influence to shape language development.

## 2. The Present Study and Hypothesis Development

We conducted a cohort study in Hong Kong that compared the language proficiency of children with long COVID symptoms to that of children who either (a) recovered fully from COVID-19 or (b) were never afflicted with COVID-19. Given the unique sociolinguistic context of Hong Kong (Bolton, 2000), where both Chinese and English are official languages, this study also allowed us to examine the differential impact of long COVID on children’s proficiency in their first (Chinese) and second languages (English). We also aimed to examine if the impacts of long COVID on language proficiency would be dependent on the school levels (i.e., kindergartens, primary schools, and secondary schools). Children’s language learning could be interfered with by the disruption of school activities and the quarantine measures during COVID-19 (Kuhfeld et al., 2020), and those interruptions for children with long COVID symptoms could be more salient. At the same time, the long COVID symptoms might plausibly impair the neurodevelopment of young children, resulting in delays or heightened difficulties in language (Johnson, 2001; Kuhl, 2004). We therefore form the following three research questions and hypotheses:

Research Question 1: Does long COVID affect children’s language proficiency, and to what extent?

Prior evidence indicates that long COVID is associated with cognitive deficits such as memory impairment and attention difficulties, which are critical for language processing (Crivelli et al., 2022; Hampshire et al., 2021). We hypothesized that children with long COVID symptoms would exhibit lower language proficiency than both children who recovered fully from COVID-19 and those who never tested positive. This hypothesis aligns with the expectation that lingering symptoms in the long COVID group would exacerbate linguistic challenges compared to the decreasing symptom severity in the COVID and no-COVID groups (Perlis et al., 2022).

**H1.**  *Children with long COVID symptoms will have lower language proficiency than those who have fully recovered from COVID-19 (H1a) and who never tested positive for COVID-19 (H1b).*

Research Question 2: Does long COVID affect children’s language proficiency differently in their first and second languages, and if so, to what extent?

In Hong Kong, English (L2) is predominantly acquired through formal schooling, whereas Chinese (L1) is reinforced through daily social interactions (Bolton, 2000). School closures and reduced face-to-face instruction during the pandemic disproportionately disrupted L2 learning environments (Kuhfeld et al., 2020). We therefore hypothesized that the negative impact of long COVID would be more pronounced in English proficiency than in Chinese, as L2 acquisition relies more heavily on structured educational settings vulnerable to pandemic-related disruptions.

**H2.**  *Long COVID will have a greater negative effect on second-language proficiency (English) than on first-language proficiency (Chinese) due to the heavier reliance on school-based learning for L2.*

Research Question 3: Does long COVID affect children’s language proficiency differently at the school level, and to what extent?

Language acquisition and cognitive development are inherently tied to educational stages. Younger children in kindergartens focus on foundational oral language skills (e.g., vocabulary and phonological awareness), while primary and secondary students progressively engage in advanced literacy tasks such as reading comprehension, critical analysis, and abstract writing (Chall, 1983; Olson & Torrance, 2009). These later stages demand sustained attention, working memory, and executive functioning—domains often impaired by long COVID’s cognitive sequelae, such as “brain fog” and fatigue (Crivelli et al., 2022; Raveendran et al., 2021). Older students may struggle more with long COVID symptoms due to complex language tasks, while younger children’s social interaction and play-based learning for foundational skill development are also affected by pandemic disruptions (Kuhfeld et al., 2020). However, due to limited prior research directly examining long COVID’s age-specific linguistic impacts, it was not feasible for us to anticipate how these school levels might differ. We thus hypothesized that the effects of long COVID on language proficiency would differ across school levels, with variations driven by distinct developmental demands and pandemic-related disruptions at each stage.

**H3.**  *The negative effects of long COVID on language proficiency will vary across different school levels.*

## 3. Method

### 3.1. Participants

Participants were recruited primarily through various social media and messaging platforms, including Facebook, Instagram, WhatsApp, and several major parent forums. Invitations were sent out across these platforms to potential participants in Hong Kong, spanning from October 2022 to May 2023. During this period, local schools transitioned back to full-time in-person classes, starting with secondary schools on 1 February 2023, and extending to primary schools and kindergartens by 15 February 2023. In March 2023, the Hong Kong government relieved the masking policy. After we ended this study (May 2023), people stopped wearing masks. This marked the end of pandemic measures in Hong Kong, combined with the widespread infection during earlier waves, making it challenging both to find uninfected individuals and to sustain interest in participation in COVID-related research. As for the duration of long COVID, various timeframes have been proposed, ranging from four weeks to three months or longer (Bliddal et al., 2021). In this study, we adopted a definition that characterized long COVID as symptoms persisting for over four weeks following the onset of acute COVID-19 illness, aligning with the Centers for Disease Control’s definition as of July 2023, which was in effect at the time our research was conducted (CDC, 2023). Consistent with earlier research, this approach allowed for the inclusion of individuals experiencing significant and potentially impactful symptoms earlier in the post-acute phase (Bliddal et al., 2021; Tleyjeh et al., 2022).

Participants included parents who (1) had children aged 3–18 years, (2) resided in Hong Kong, (3) were proficient in reading and writing Chinese, and (4) had children without any known language-related deficiencies such as dyslexia. From 1542 eligible participants, we used stratified sampling to ensure representativeness across COVID-19 infection status, children’s age group (K-12), gender, and region of residence (across 18 administrative districts in Hong Kong).

The final study included 1244 parent–child dyads. Boys comprised 53.5% of the sample. The detailed demographic characteristics of the child sample stratified by COVID-19 symptoms and school level are stated in Table 1.

Regarding household monthly income, 33.4% of parents reported earning HKD 20,001–40,000 monthly, which reflects the average financial situation in Hong Kong (Census and Statistics Department, 2021). Educational levels varied, with most having completed secondary education (47.2%), followed by bachelor’s degrees (22.7%). Overall, the sample was diverse and representative of the general population in Hong Kong, ensuring the robustness and generalizability of the findings.

### 3.2. Procedures

This study received ethical approval from the institution of the first author. Before participation, parents were provided with information sheets detailing the study and signed informed consent forms on behalf of themselves and their children. The main survey, which took approximately 20 min to complete, was filled out by the parents. As a token of appreciation for their participation, each participating family received HKD 50 (approximately USD 6.4).

### 3.3. Measurements

Language Proficiency. Children’s language proficiency was measured using an adapted Language Experience and Proficiency Questionnaire (LEAP-Q), which evaluates bilingual and multilingual language profiles and has been validated in various languages, including Chinese and English (Kaushanskaya et al., 2020). This adaptation required participants to assess their proficiency in Chinese and English across four domains, speaking, listening, reading, and writing, using an 11-point scale from 0 (no proficiency) to 10 (mastery). As Cantonese and Mandarin are two spoken dialects of Chinese that Hong Kong students may learn in local schools, we asked the participants to give separate ratings of (1) speaking and (2) listening for these two dialects. The Cronbach’s alphas of the overall language proficiency scores (α = 0.91) and the subdimensions of speaking (α = 0.68), listening (α = 0.68), reading (α = 0.75), and writing (α = 0.85) were satisfactory.

COVID-19 Status. We developed questions to categorize the children’s COVID-19 status. The construction of these items followed the corresponding measures from previous studies (Buttery et al., 2021; Whitaker et al., 2022), and the type of symptoms of long COVID accepted in the literature (Borch et al., 2022; Perlis et al., 2022). The first part inquired about the students’ history of COVID-19 diagnoses, including the number of diagnoses, the most recent date of diagnosis, the method of diagnosis, the variant of the virus, and vaccination status at the time of diagnosis. The second part was exclusive to children who had a history of COVID-19. We gathered information regarding whether they had any persistent long COVID symptoms, whether the symptoms were diagnosed by medical professionals, how long the symptoms persisted, and whether the symptoms were cured (CDC, 2023). Based on their responses, the participants were sorted into three categories: (1) long COVID (i.e., children who had long COVID symptoms for over four weeks) (Aiyegbusi et al., 2021; CDC, 2023; Gutzeit et al., 2024); (2) COVID (i.e., children who recovered fully from COVID-19 and did not have any long COVID symptoms at present); and (3) no-COVID (i.e., children who never tested positive for COVID-19).

### 3.4. Data Analysis

To test H1, we applied a one-way ANOVA to examine whether the language proficiency was different between the long COVID group, COVID group, and no-COVID group. Scheffe’s post hoc tests were employed to examine if the language proficiency of the long COVID group was significantly lower than that of the COVID group (H1a) and no-COVID group (H1b). For H2, we first conducted a one-way ANOVA to unpack the effect of long COVID on first- and second-language proficiency (i.e., Chinese and English). We also conducted a two-way repeated-measures ANOVA where COVID-19 status and language (i.e., first language vs. second language) were the between-subject and within-subject factors, respectively. For H3, we conducted a two-way ANOVA to examine if the findings of H1 were consistent between children at different school levels (i.e., kindergarten, primary school, and secondary school). Separate ANOVAs were employed for the overall language proficiency and its subdimensions (i.e., speaking, listening, reading, and writing).

Post hoc comparisons were conducted using Scheffé’s method, a conservative approach that controls the family-wise error rate (FWER) for all pairwise comparisons. This method was selected for its robustness to unequal group sizes and variances, ensuring stringent protection against Type I errors in our multi-factorial analyses (Maxwell et al., 2017; Scheffe, 1953). Statistical Package for the Social Sciences (SPSS) 29 was used for the analysis, controlling for children’s age, sex, parental education, and monthly income.

## 4. Results

### 4.1. Data Screening

The descriptive statistics and tests of normality for the main variables, including language proficiency and its subdimensions, were analyzed. The mean scores were 5.05 (*SD* = 1.69) for kindergarten, 6.21 (*SD* = 1.86) for primary, and 6.64 (*SD* = 1.87) for secondary students. Table 2 shows the descriptive information about the children’s language proficiency scores. Normality tests showed that the data were generally normally distributed. The overall language proficiency score and its subdimensions exhibited acceptable ranges of skewness (−0.011 to −0.589) and kurtosis (−2 to +2). Kolmogorov–Smirnov and Shapiro–Wilk tests both showed that the data were not significantly different from normality (*p* > 0.05). Therefore, parametric statistical tests (i.e., ANOVA) were appropriate.

### 4.2. Effects of Long COVID on Language Proficiency (RQ1)

Language Proficiency—Overall Score. One-way ANOVA revealed a significant main effect of COVID-19 status (*F*(2, 1241) = 9.039, *p* < 0.001, *η*^2^ = 0.014). The long COVID group exhibited significantly lower overall language proficiency than the COVID group and the no-COVID group (*ps* < 0.001). The difference between the COVID and no-COVID groups was not statistically significant. The patterns support H1a and H1b.

Language Proficiency—Subdimensions. Separate one-way ANOVAs on subdimensions suggested significant main effects of COVID-19 status on speaking (*F*(2, 1241) = 14.221, *p* < 0.001, *η*^2^ = 0.022), listening (*F*(2, 1241) = 15.028, *p* < 0.001, *η*^2^ = 0.024), and reading proficiency (*F*(2, 1241) = 3.843, *p* = 0.022, *η*^2^ = 0.006), but not on writing (*F*(2, 1241) = 0.602, *p* = 0.548, *η*^2^ = 0.001). Post hoc analysis using the Scheffe method revealed that the long COVID group scored significantly lower than the COVID group on speaking and listening (*ps* < 0.001), and they also demonstrated poorer reading compared to the COVID group, which was marginally significant (*p* = 0.053). The long COVID group differed significantly from the no-COVID group in speaking, listening, and reading (*ps* < 0.05). For all subdimensions, the differences between the COVID and no-COVID groups were insignificant. Table 1 presents the results.

### 4.3. Effect of Long COVID by Language (RQ2)

A two-way ANOVA showed a significant main effect of language (*F*(1, 1241) = 682.193, *p* < 0.001, *η_p_*^2^ = 0.355), indicating overall higher language proficiency in Chinese (L1) than English (L2), *p* < 0.001. The main effect of COVID-19 status (*F*(2, 1241) = 9.044, *p* < 0.001, *η_p_*^2^ = 0.014) on language proficiency was also significant. However, contrary to H2, the interaction between language and COVID-19 status was not significant.

Sub-group analysis showed that the patterns of results were similar between the proficiency of Chinese and English. Significant main effects of COVID-19 status were evidenced in the overall language proficiency of both Chinese and English, consistently showing that the long COVID group scored significantly lower than the two other groups (*ps* < 0.05). Lower proficiency in speaking and listening of both languages was also observed in the long COVID group, as compared to the two other groups (*ps* < 0.05). A significant group difference in COVID-19 status on reading was found in English, but not in Chinese. Both languages did not reveal a significant group difference in COVID status for writing.

No significant differences were observed between the COVID and no-COVID groups. The overall pattern of the results did not support H2 (see Table 3).

### 4.4. Effects of Long COVID Status by School Level (RQ3)

A two-way ANOVA revealed a significant main effect of COVID-19 status (*F*(2, 1231) = 4.955, *p* = 0.007, *η_p_^2^* = 0.008) and school levels (*F*(2, 1231) = 85.45, *p* < 0.001, *η_p_^2^* = 0.122) on language proficiency. Furthermore, the interaction between COVID-19 status and school level showed a marginal significance (*F*(4, 1231) = 2.151, *p* = 0.072, *η*^2^ = 0.007), suggesting that the effect of COVID-19 status on language proficiency may vary by school level.

We then conducted separate one-way ANOVAs to unpack the effects at each school level. For kindergarten children, there was no significant effect of COVID-19 status on overall language proficiency or subdimensions. Primary school children exhibited significant main effects of COVID-19 status on overall language proficiency (*F* = 7.526, *p* < 0.001, *η*^2^ = 0.027), speaking (*F* = 7.389, *p* < 0.001, *η*^2^ = 0.026), listening (*F* = 8.668, *p* < 0.001, *η*^2^ = 0.031), reading (*F* = 4.276, *p* = 0.014, *η*^2^ = 0.015), and writing (*F* = 5.302, *p* = 0.005, *η*^2^ = 0.019). The long COVID group reported significantly lower scores across all these domains compared to both the COVID and no-COVID groups.

Secondary school students demonstrated even more pronounced deficits, with significant effects on overall proficiency (*F* = 9.973, *p* < 0.001, *η*^2^ = 0.065), speaking (*F* = 10.673, *p* < 0.001, *η*^2^ = 0.069), listening (*F* = 12.497, *p* < 0.001, *η*^2^ = 0.080), and reading (*F* = 10.156, *p* < 0.001, *η*^2^ = 0.066). Writing proficiency did not differ significantly between groups (*F* = 0.602, *p* = 0.548). H3 was partially supported. No significant differences were observed between the COVID and no-COVID groups (see Table 4, Table 5 and Table 6 for detailed results).

## 5. Discussion

Although the negative impacts of COVID-19 on children’s development have been extensively researched, how long COVID symptoms are associated with children’s language proficiency remains unknown (Fainardi et al., 2022). Using a sample of 1244 participants from Hong Kong, our results showed that children affected by long COVID symptoms demonstrated lower language proficiency as compared to children in the COVID and no-COVID conditions. These findings align with the prior literature about the potential impacts of long COVID on cognitive–linguistic abilities in adults (Cummings, 2023, 2024), providing initial evidence that the negative consequences of long COVID may also manifest in the pediatric and adolescent populations.

### 5.1. Substantiated Impact of Long COVID on Speaking and Reading Proficiency

Our study found that long COVID symptoms may have considerable negative effects on children’s language proficiency, aligning with other research regarding long COVID’s impact on children’s developmental outcomes (Ahrens et al., 2021; Loades et al., 2020). Children with long COVID tend to have difficulties in memory, attention, and executive function—domains integral to language processing and acquisition—thus impeding expressive and receptive language skills (Elbro et al., 2011). Similarly, memory issues caused by long COVID symptoms may also limit vocabulary retention and word recall, while attention deficits can obstruct the focus on and processing of language (Stavridou et al., 2020). Our study’s findings suggest that the language impairments in children with long COVID symptoms might go beyond the social and educational disruptions caused by the pandemic (Charney et al., 2021). It is important to note that the participants in our study experienced lockdowns, school closures, and other quarantine measures during the study period. These findings indicate that the language impairments observed in children with long COVID are likely a direct consequence of the condition, rather than solely the result of pandemic-related disruptions (Loades et al., 2020). In summary, the cognitive impairments stemming from long COVID, along with prolonged isolation, collectively represented a significant risk to the normal pattern of language development in children.

### 5.2. Reduced Proficiency of Long COVID Group in Both First and Second Languages

Our findings revealed that long COVID symptoms significantly impair children’s proficiency in both their L1 (Chinese) and L2 (English), with no observable distinction between the effects on either language. This pattern of results contradicts the assumption that L2 would be more vulnerable to the negative impact of long COVID symptoms because it is supposed to be more reliant on formal education systems. Considering the bilingual setting of Hong Kong, the regular use of English outside of academic settings might have instilled a level of resilience in language proficiency that could counteract the effects of long COVID (Bolton, 2000). Therefore, our study’s findings may not be universally generalizable. This premise invites further research in other bilingual or multilingual contexts to determine whether such protective effects against long COVID’s language impact are present elsewhere, and to understand whether language impairment due to long COVID is independent of whether the language is learned naturally or through formal education.

### 5.3. Different Impacts of Long COVID on Language Proficiency Across School Levels

The impact of long COVID on language skills appeared to be more pronounced in older students, with secondary school students with long COVID symptoms exhibiting the most significant language deficits. These findings may be attributed to their developmental stages. Primary years are the period when children learn to become proficient speakers and skilled readers through structured learning; their rapid development in language and reading has the potential to buffer the negative impacts of long COVID on language proficiency; therefore, the negative impact is not detectable. At the same time, young children’s language development can be well supported by their parents at home and does not yet heavily rely on formal instruction (Chan et al., 2015). As children move into late primary and secondary school, they transition from “learning to read” to “reading to learn”, as per Chall’s stages of reading development (Tan et al., 2022). They have established language skills well before and are at the stage of consolidating and strengthening their high-level language capacities, such as complex syntax, advanced vocabulary, and abstract language concepts, through formal education in school (Chall, 1983; Olson & Torrance, 2009). The cognitive demands of language tasks increase significantly during this stage, as students are often expected to utilize their language skills for higher-order thinking, critical analysis, and learning across content areas. Since their fundamental language development has plateaued, disruptions caused by COVID-19 can significantly affect their engagement with the curriculum and impede academic progress. Consequently, the language proficiency of older children is more likely to be compromised by prolonged confinement and limited social interaction due to long COVID than that of younger children. This pattern highlights the potential for long COVID to considerably impair the linguistic abilities of older children, underscoring the importance of ongoing cognitive assessments and age-appropriate intervention, particularly concerning language proficiency. Our findings provide new evidence from children at different educational levels to the literature, which was previously dominated by evidence from adults (Cummings, 2023). It is important to adopt a developmental perspective when considering the impact of long COVID on children. This perspective recognizes the different stages of language development and the varying vulnerabilities at each stage, emphasizing the need for age-appropriate interventions and support designed to mitigate the adverse effects of long COVID on language development.

In particular, secondary school students face significant challenges in advanced language skills (e.g., abstract writing, critical analysis). Educators are advised to adopt comprehensive support strategies, such as implementing executive function training programs focusing on working memory and sustained attention (Cummings, 2023), alongside curricular adaptations such as extended assignment deadlines, breaking down complex tasks into step-by-step instructions, and utilizing visual aids to enhance comprehension. Moreover, primary school children may benefit from structured phonic reinforcement and small-group activities to rebuild vocabulary and listening comprehension disrupted by pandemic learning gaps (Kuhfeld et al., 2020). Lastly, while kindergarteners showed no significant deficits in our study, proactive monitoring through routine language screenings can ensure early detection of delayed symptom onset. For all groups, collaboration between educators, speech–language pathologists, and pediatricians is critical to tailor interventions—such as bilingual support strategies leveraging L1 (Cantonese) to scaffold L2 (English) proficiency (Bolton, 2000)—to the unique sociolinguistic and cognitive needs of Hong Kong children recovering from long COVID. These approaches not only address immediate deficits but also foster resilience against long-term academic setbacks.

### 5.4. Limitations and Future Directions

This study has several limitations. First, due to the rapid progress of the pandemic and for practical reasons, it was not feasible for us to measure our participants’ language proficiency before their infection with COVID-19. The absence of pre-COVID-19 language proficiency data precludes definitive conclusions about long COVID’s direct impact. Without baseline measurements, we cannot rule out pre-existing differences in language skills between the groups. We have done our best to minimize this constraint. Our stratified sampling ensured representativeness across socioeconomic and demographic factors (e.g., parental education, income), and we statistically controlled for age and school level to mitigate the plausible confounding effects. Second, our reliance on self-reported measures might not only impair the objectiveness of the assessment but also reduce the sensitivity in detecting nuanced decrements in linguistic capabilities (Ahrens et al., 2021; Ganesan et al., 2021). Future research should incorporate objective, standardized assessments that are sensitive to these foundational aspects of language function (Macbeth et al., 2022). Moreover, the cross-sectional design also limits our understanding of the progression or persistence of language deficits over time. Additionally, while this research has taken into account the presence of long COVID, the diversity of symptoms and their individual effects on language were not controlled for due to the limitations of our sample size. In this research, we considered the presence of long COVID, defined here as the persistence of one or more long COVID symptoms for at least four weeks following the onset of acute COVID-19 illness. However, this 4-week timeframe, while capturing potentially significant early symptoms, may include individuals whose symptoms ultimately resolve and differs from the more widely adopted 12-week timeframe (Aiyegbusi et al., 2021). This variability in definitions across studies makes direct comparisons challenging and highlights the need for standardized definitions. Future studies with larger cohorts and a longitudinal design are needed to discern patterns in how varying long COVID symptomatology may differentially impact language proficiency in the long term. To advance this emerging field, we propose several directions for future investigations. First, longitudinal cohort studies should track language proficiency for an extended period (e.g., 3–5 years) post-diagnosis, incorporating baseline data to disentangle acute versus chronic effects. Second, to address the limitations of self-reported measures, future studies can consider employing standardized linguistic tests (e.g., Comprehensive Assessment of Spoken Language, CASL-2 (Rehfeld & Padgett, 2019); Expressive Vocabulary Test, (Roberts et al., 2007)) that objectively quantify specific language subdomains. Finally, machine learning approaches could delineate how specific symptom clusters (e.g., fatigue–brain fog dyads) influence language outcomes, enabling personalized therapeutic strategies. These future directions may advance the current understanding of the relationship between long COVID and the language development of children, and how targeted interventions may support affected children.

## 6. Conclusions

Our study offers the first evidence of the multifaceted impact of long COVID on the language proficiency of children from different educational levels. The findings revealed a marked impairment in language skills, particularly in speaking and listening, among children suffering from long COVID compared to their peers who had either fully recovered from COVID-19 or were never infected. This impairment was observed across both the first and second languages, indicating that long COVID can disrupt multiple facets of language development. Our results also showed that the impact of long COVID on language development varies with school levels, with primary and secondary school students demonstrating particularly notable deficits in language proficiency following long COVID. These findings suggest that long COVID may have disrupted the acquisition and refinement of crucial language skills during a critical period of cognitive and academic growth. Future research should continue to investigate the effects of long COVID on various aspects of students’ development, academic performance, and well-being, taking into account the potential interactions between COVID-19 status and other factors, such as school levels, age, and individual differences.

## Figures and Tables

**Table 1:** Demographic characteristics of the sample of children.

Sample Characteristics			
Total Sample	*N* = 1244, *M_age_* = 8.75 ± 4.27; Boys, 53.5%		
COVID-19 Symptoms	Long COVID group *N* = 353, 28.4% *M_age_* = 9.21 ± 4.42	COVID group *N* = 480, 38.6% *M_age_* = 8.61 ± 4.16	No-COVID group *N* = 411, 33.0% *M_age_* = 8.51 ± 4.21
School Levels	Kindergarten *N* = 408, 32.8% *M_age_* = 4.42 ± 1.26	Primary school *N* = 547, 44.0% *M_age_* = 9.69 ± 1.96	Secondary school *N* = 289, 23.2% *M_age_* = 14.97 ± 1.85

**Table 2:** Descriptive language proficiency scores for different school levels and COVID symptoms.

Mean (SD)	Overall Score	Speaking	Listening	Reading	Writing
School Levels					
Kindergarten	5.05 (1.69)	5.54 (1.76)	5.83 (1.80)	4.57 (2.36)	3.59 (2.43)
Primary School	6.21 (1.86)	6.33 (1.99)	6.58 (1.93)	6.13 (2.06)	5.56 (2.05)
Secondary School	6.64 (1.87)	6.68 (1.97)	6.89 (2.03)	6.61 (2.12)	6.26 (2.03)
COVID-19 Status					
Long COVID	5.57 (1.90)	5.69 (1.99)	5.93 (2.02)	5.44 (2.28)	4.96 (2.38)
COVID	6.10 (1.90)	6.38 (1.89)	6.63 (1.85)	5.83 (2.36)	5.13 (2.50)
No-COVID	6.04 (1.92)	6.28 (1.96)	6.55 (1.96)	5.85 (2.31)	5.11 (2.37)

**Table 3:** Mean differences in language scores between children with long COVID and no long COVID using multiple comparisons.

Language Proficiency	Variable 1	Variable 2	Mean Difference	Std. Error	Sig.	95% Confidence Interval	
						Lower Bound	Upper Bound
Overall Scores	Long COVID	COVID	−0.531 *	0.134	<0.001	−0.859	−0.204
		No-COVID	−0.479 *	0.138	0.003	−0.818	−0.140
Speaking	Long COVID	COVID	−0.691 *	0.136	<0.001	−1.026	−0.357
		No-COVID	−0.592 *	0.141	<0.001	−0.939	−0.247
Listening	Long COVID	COVID	−0.699 *	0.136	<0.001	−1.032	−0.367
		No-COVID	−0.622 *	0.141	<0.001	−0.967	−0.278
Reading	Long COVID	COVID	−0.395	0.163	0.053	−0.794	0.004
		No-COVID	−0.414 *	0.168	0.049	−0.828	−0.002
Writing	Long COVID	COVID	−0.174	0.170	0.592	−0.591	0.243
		No-COVID	−0.158	0.176	0.668	−0.590	0.273

Note: * indicates significance at the *p* < 0.05 level. Long COVID stands for children with long COVID symptoms. COVID stands for children who have recovered from COVID-19 without long COVID symptoms. No-COVID stands for children who have never had COVID-19.

**Table 4:** Effect of long COVID by language types.

Language Type	Measure	*F*-Value	*p*-Value	*η* ^2^
Chinese (First Language)	Overall Proficiency	5.62	0.004	0.009
	Speaking	15.504	<0.001	0.024
	Listening	19.99	<0.001	0.031
	Reading	6.535	0.091	0.005
	Writing	0.828	0.437	0.001
English (Second Language)	Overall Proficiency	9.565	<0.001	0.015
	Speaking	10.103	<0.001	0.016
	Listening	8.697	<0.001	0.014
	Reading	3.96	0.019	0.006
	Writing	0.398	0.672	0.001

**Table 5:** Mean differences in language scores between children with long COVID and no long COVID across first language (Chinese) and second language (English) using multiple comparisons.

Language Proficiency	Variable 1	Variable 2	Mean Difference	Std. Error	Sig.	95% Confidence Interval	
						Lower Bound	Upper Bound
Chinese Overall Scores	Long COVID	COVID	−0.462 *	0.157	0.014	−0.847	−0.076
		No-COVID	−0.485 *	0.162	0.012	−0.884	−0.086
Chinese Speaking	Long COVID	COVID	−0.855 *	0.161	<0.001	−1.250	−0.459
		No-COVID	−0.730 *	0.167	<0.001	−1.139	−0.321
Chinses Listening	Long COVID	COVID	−0.934 *	0.154	<0.001	−1.313	−0.556
		No-COVID	−0.780 *	0.159	<0.001	−1.171	−0.388
Chinese Reading	Long COVID	COVID	−0.357	0.186	0.159	−0.81	0.10
		No-COVID	−0.370	0.193	0.158	−0.84	0.10
Chinese Writing	Long COVID	COVID	−0.237	0.185	0.440	−0.690	0.220
		No-COVID	−0.153	0.191	0.726	−0.620	0.320
English Overall Scores	Long COVID	COVID	−0.596 *	0.1431	<0.001	−0.946	−0.245
		No-COVID	−0.508 *	0.1481	0.003	−0.8717	−0.145
English Speaking	Long COVID	COVID	−0.664 *	0.166	<0.001	−1.072	−0.260
		No-COVID	−0.677 *	0.172	<0.001	−1.103	−0.260
English Listening	Long COVID	COVID	−0.640 *	0.171	<0.001	−1.065	−0.220
		No-COVID	−0.641 *	0.177	0.001	−1.070	−0.210
English Reading	Long COVID	COVID	−0.434 *	0.177	0.050	−0.874	0.000
		No-COVID	−0.459 *	0.183	0.043	−0.911	−0.015
English Writing	Long COVID	COVID	−0.111	0.180	0.826	−0.552	0.332
		No-COVID	−0.163	0.186	0.680	−0.623	0.291

Note: * indicates significance at the *p* < 0.05 level. Long COVID stands for children with long COVID symptoms. COVID stands for children who have recovered from COVID-19 without long COVID symptoms. No-COVID stands for children who have never had COVID-19.

**Table 6:** Mean differences in language scores between children with long COVID and no long COVID across different school levels using multiple comparisons.

School Levels	Language Proficiency	Variable 1	Variable 2	Mean Difference (I-J)	Std. Error	Sig.	95% Confidence Interval	
							Lower Bound	Upper Bound
Kindergarten	Overall scores	Long COVID	COVID	−0.036	0.210	0.985	−0.554	0.481
			No-COVID	−0.061	0.218	0.961	−0.596	0.474
	Speaking	Long COVID	COVID	−0.307	0.219	0.376	−0.844	0.231
			No-COVID	−0.375	0.226	0.254	−0.931	0.181
	Listening	Long COVID	COVID	−0.253	0.224	0.529	−0.804	0.298
			No-COVID	−0.175	0.232	0.752	−0.744	0.394
	Reading	Long COVID	COVID	0.216	0.293	0.762	−0.504	0.936
			No-COVID	0.213	0.303	0.781	−0.532	0.958
	Writing	Long COVID	COVID	0.441	0.302	0.344	−0.300	1.183
			No-COVID	0.305	0.312	0.620	−0.461	1.072
Primary School	Overall scores	Long COVID	COVID	−0.700	0.195	0.002	−1.178	−0.222
			No-COVID	−0.653 *	0.202	0.006	−1.151	−0.157
	Speaking	Long COVID	COVID	−0.779 *	0.209	0.001	−1.292	−0.267
			No-COVID	−0.620 *	0.217	0.017	−1.153	−0.088
	Listening	Long COVID	COVID	−0.757 *	0.202	<0.001	−1.253	−0.262
			No-COVID	−0.753 *	0.210	0.002	−1.268	−0.240
	Reading	Long COVID	COVID	−0.553 *	0.217	0.040	−1.086	−0.020
			No-COVID	−0.589 *	0.225	0.033	−1.143	−0.037
	Writing	Long COVID	COVID	−0.641 *	0.215	0.012	−1.171	−0.113
			No-COVID	−0.618 *	0.224	0.023	−1.167	−0.069
Secondary School	Overall scores	Long COVID	COVID	−1.105 *	0.259	<0.001	−1.744	−0.467
			No-COVID	−0.890 *	0.267	0.004	−1.547	−0.235
	Speaking	Long COVID	COVID	−1.209 *	0.272	<0.001	−1.880	−0.539
			No-COVID	−0.950 *	0.280	0.004	−1.639	−0.262
	Listening	Long COVID	COVID	−1.333 *	0.280	<0.001	−2.022	−0.644
			No-COVID	−1.081 *	0.288	0.001	−1.789	−0.374
	Reading	Long COVID	COVID	−1.185 *	0.294	<0.001	−1.910	−0.461
			No-COVID	−1.152 *	0.302	<0.001	−1.897	−0.409
	Writing	Long COVID	COVID	−0.530	0.289	0.188	−1.242	0.182
			No-COVID	−0.253	0.297	0.697	−0.984	0.478

Note: * indicates significance at the *p* < 0.05 level. Long COVID stands for children with long COVID symptoms. COVID stands for children who have recovered from COVID-19 without long COVID symptoms. No-COVID stands for children who have never had COVID-19.

## Data Availability

The data necessary to reproduce the analyses presented here are not publicly accessible.
